# Anatomic and functional leg-length inequality: A review and recommendation for clinical decision-making. Part I, anatomic leg-length inequality: prevalence, magnitude, effects and clinical significance

**DOI:** 10.1186/1746-1340-13-11

**Published:** 2005-07-20

**Authors:** Gary A Knutson

**Affiliations:** 1 840 W. 17^th^, Suite 5 Bloomington, IN, 47404, USA

**Keywords:** Leg-length inequality, anatomic, back pain, chiropractic

## Abstract

**Background:**

Leg-length inequality is most often divided into two groups: anatomic and functional. Part I of this review analyses data collected on anatomic leg-length inequality relative to prevalence, magnitude, effects and clinical significance. Part II examines the functional "short leg" including anatomic-functional relationships, and provides an outline for clinical decision-making.

**Methods:**

Online database – Medline, CINAHL and MANTIS – and library searches for the time frame of 1970–2005 were done using the term "leg-length inequality".

**Results and Discussion:**

Using data on leg-length inequality obtained by accurate and reliable x-ray methods, the prevalence of anatomic inequality was found to be 90%, the mean magnitude of anatomic inequality was 5.2 mm (SD 4.1). The evidence suggests that, for most people, anatomic leg-length inequality does not appear to be clinically significant until the magnitude reaches ~ 20 mm (~3/4").

**Conclusion:**

Anatomic leg-length inequality is near universal, but the average magnitude is small and not likely to be clinically significant.

## Review

Leg-length inequality (LLI) is a topic that seemingly has been exhaustively examined; yet much is left to be understood. Reviews by Mannello [1] and Gurney [2] on leg-length inequality and Cooperstein and Lisi on pelvic torsion [3] are highly recommended as sources to provide expanded and longer time-frame background information on this topic. The information provided by these authors, however extensive, is incomplete relative to clinical decision-making. Further, several questions have remained largely unanswered regarding anatomic leg-length inequality and the so-called functional short leg, or more accurately, unloaded leg-length alignment asymmetry (LLAA). These include: how common is anatomic LLI, what is the average amount of anatomic LLI, what are the effects of anatomic LLI, how much anatomic LLI is necessary to be clinically significant, and what are the incidental and functional relationships of anatomic LLI to unloaded leg-length alignment asymmetry? The purpose of this review is to highlight current research to answer these questions and help in clinical decision-making.

### Methods

In the 1970's studies began to show that clinical measurements of LLI were inaccurate and the use of x-ray, controlling for magnification and distortion, was necessary [4-6]. By 1980 the accuracy of the measurements with the standing x-ray had been established, with Friberg then demonstrating reliability of the method on subjects [7]. For these reasons, this review starts in the 1970's with studies that used the reliable x-ray procedure as described by Friberg.

To answer the question regarding the prevalence of anatomic leg-length inequality, Medline, CINAHL, MANTIS and library searches (using key words "leg-length inequalty") were performed for studies done from 1970–2005. Studies which did not describe, or use the reliably precise radiographic method, or that did not provide their LLI measurement data, were excluded.

### Prevalence of anatomic leg-length inequality

**Figure 1:**
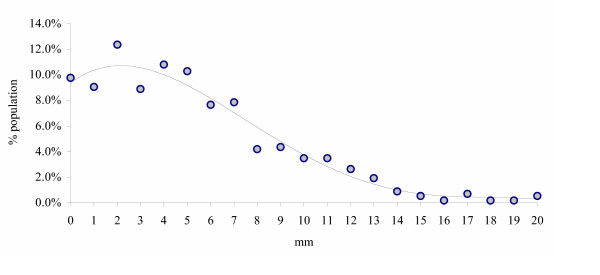
"Incidence" of anatomic leg-length inequality magnitude.

**Figure 2:**
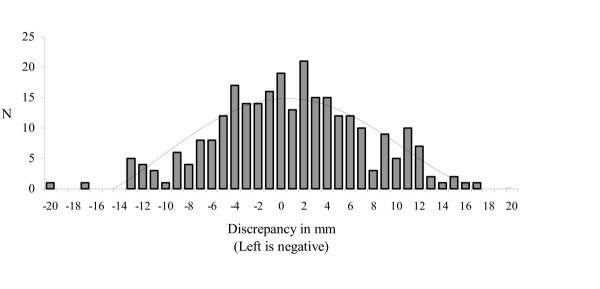
Magnitude of anatomic leg-length inequality; right vs. left.

Several studies using the precise radiographic method (Table 1) contained data, which quantified LLI in incremental millimetric measurements [8-15]. These studies were combined giving a population of n = 573, with a LLI range of 0–20 mm. The mean LLI was 5.21 mm (SD 4.1 mm) or approximately 3/16". The results of these studies are shown in Figure 1. Six of the studies, with combined population of n = 272, broke their data down into right or left LLI [8-12,14]. Figure 2 shows those results; note the curve is shifted slightly towards leg-length discrepancy on the right. This finding – that the right leg is anatomically shorter more often – is consistent with other studies that have found the left leg to be anatomically longer 53–75% of the time [6,7,9]. Using the same studies [8-12,14] to compare the magnitude of the discrepancy of right (n = 140) and left (n = 114) legs finds only a 0.84 mm difference, which is not statistically significant (p = 0.08, t-test). This means that while the right leg is anatomically short more often, the amount of the discrepancy is no greater than a short left leg.

**Table 1:** Studies using reliable means of determining magnitude of anatomic leg-length inequality

Study	Population	"N" (573)	Subjects/Notes	Controls	Av LLI (SD)
Gross R. 1983	Male marathon runners, age 24–49	33	No deleterious effect of the LLI		4.9 mm (3.8)
Venn et al 1983	Randomly chosen patients	60			5.4 mm (4.0)
Cleveland et al 1988	Low back pain patients	10	Standing and supine x-ray		4.7 mm (5.8)
Hoikka et al 1989	Chronic low back pain patients	100			4.9 mm (3.6)
Beattie et al 1990	Clinical subjects, age 22–60	19	10 with history of LLI or lower extremity or back pain	9 healthy	6.8 mm (5.7)
Soukka et al 1991	Four defined occupational and gender groups, age 35–54	247	194 with prior back pain (>12 mo ago and during last 12 mo with and without disability)	53 who never had back pain	5.0 mm (3.9)
Rhodes et al 1995	New LBP patients Chiropractic practice	50	Age 18–40 26 men 24 women		6.3 mm (4.1)
Mincer et al 1997	Volunteers	54	no history of back pain in last 6 months 10 men 44 women		2.4 mm (1.8)

Four of the radiographic studies [8,10,12,15] identified measured LLI subjects by gender (n = 116). There was no difference (p = 0.87, t-test) between male and female LLI as shown in Table 2, suggesting that gender plays little role in the amount of anatomic LLI. One study [12] provided data on subject height (n = 19), which was plotted against LLI giving only a fair correlation coefficient of 0.31. However, Soukka et al, using a much larger number of subjects (n = 247) did find a correlation between height and LLI (p = 0.02)[13]. Men, being taller than women on average, would be expected to show a larger LLI, but did not. The discrepancy in these data is difficult to explain.

**Table 2:** Relationship between gender and anatomic leg-length inequality

LLI and Gender (Refs 8,10,12,15)	N	Mean LLI (mm)
Male	58	5.1 (4.3)
Female	58	5.2 (4.6)
		P = 0.87 (t-test)

Seven of the studies identified subjects with LLI as being symptomatic (n = 347) or asymptomatic (n = 165) [8-10,12-15]. Symptoms included a variety of kinetic chain (knee, hip) problems and low back pain. Asymptomatic was variously defined from no complaints, to no back pain in the last six months [15], to no low back pain in the last 12 months [13]. Symptomatic subjects had a mean LLI of 5.1 mm (SD 3.9); asymptomatic subjects had a mean LLI of 5.2 mm (SD 4.2). There is no statistical difference in the LLI between these two groups (p = 0.75, t-test). The mean LLI for these groups is virtually identical to the overall combined mean, suggesting that the average LLI is not correlated to symptomatic problems, especially low back pain.

**Figure 3:**
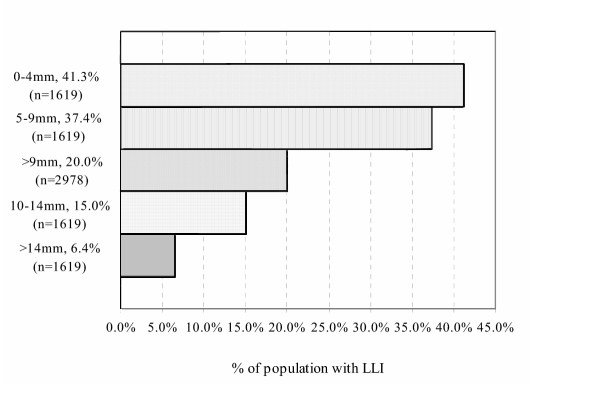
Ranges of anatomic leg-length inequality.

Recognizing that measurements to the precision of a millimeter will be prone to error, other studies – again, using precise radiographic methods – have examined LLI within a measured range [7,16,17]. These findings, combined with the millimetric measure studies, are noted in Figure 3, and provide an even larger pool of data for LLI. This data table shows, for example, that in a pooled population of 2,978 people, 20.1% had a LLI of 10 mm or more. Collecting x-ray data from 421 subjects with low back pain from an osteopathic manipulative practice, Juhl et al [18] reported on the incidence of leg-length and sacral base unleveling. The data from Juhl et al indicated that 43% of those examined had LLI of 10 mm or more, twice the rate noted from the pooled data in this review. A significant difference of Juhl et al's methods of examination was that the central ray was directed at the level of the sacral base, and not the femoral heads. Due to this methodological difference, lack of reported reliability of this method, and the significant disagreement with others as to incidence, the data from Juhl et al regarding the incidence of anatomic leg-length inequality was not used.

Using the data from the millimetric measurement, 90% of the population has some anatomic leg-length asymmetry. This finding is in accord with other studies [19,20]. Larger LLI – more than 20 mm (~ 3/4") – was calculated in a population of 2.68 million, to be 1 in 1000 [21]. References will be made later in this paper to the data compiled in these two tables.

Finally, in a retrospective study of 106 consecutive patients, Specht and De Boer report on the use of 14" × 36" x-ray films to determine LLI [22]. This x-ray method, less reviewed than the methods noted above, does not direct the central ray at the femoral heads and therefore uses a mathematical formula to take the effect innominate rotation into account in measuring LLI. The results calculated from the data presented showed an average LLI of 5.5 mm (SD 3.9), which is nearly identical to the multi-study average noted above.

### Effects of LLI

**Figure 4:**
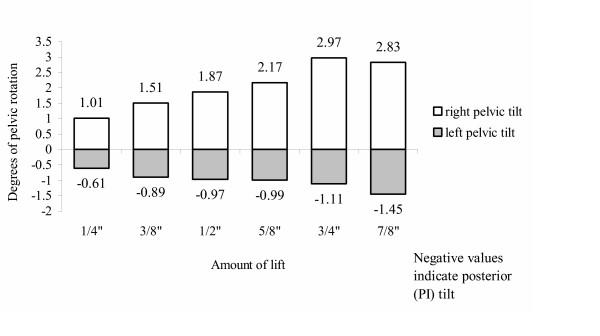
Pelvic rotation with left heel lift (Cummings).

The most common effect of anatomic LLI is rotation of the pelvis and/or innominate bones – often referred to as pelvic torsion – in the sagittal and/or frontal planes [3,23-25]. Mechanically, in the standing position, the weight of the body in the pelvis induces a force vector through the hip joints and towards the feet. With asymmetry of the leg-lengths, the pelvis, being pushed down on the femoral heads, must rotate or torsion. The innominate movement tends to be anterior on the side of the anatomically short leg and posterior contralaterally [23,26]. In studies of pelvic rotation imposed by foot lifts, there was an approximately linear relationship in pelvic torsion as the leg was lengthened from 1/4 to 7/8" [23]. A chart, based on the work of Cummings et al, shows the degrees of torsion relative to lengthening of the left leg (Figure 4). Note that the artificial lengthening of the left leg caused more rotation of the contralateral hemipelvis in an anterior direction – the short leg side – than posterior rotation ipsilaterally.

The relationship of LLI to pelvic torsion is supported by the data of others [27]. Walsh et al [24] found that pelvic obliquity was the most common method of compensating for LLI up to 22 mm. With larger amounts of leg-length inequality, subjects begin to develop flexion of the knee in the long leg [24]. While the degree of pelvic torsion due to the imposition of lifts tends to be linear, there are many factors – including innominate asymmetry, freedom of SI joint movement, and hypertonic suprapelvic muscles – that can affect pelvic torsion. Several authors emphasize that it is a mistake to assume that the side and amount of LLI can be reliably deduced from pelvic crest unleveling [17,26,28].

Other effects of LLI and pelvic torsion have been demonstrated by Giles et al [29,30]. These compensations include alterations and asymmetry of lumbosacral facet joint angles, postural scoliosis, concavities in the vertebral body end-plates, wedging of the 5 th lumbar vertebra and traction spurs. However, no relationship of these findings to symptoms was claimed.

Along the lines of symptomatic problems associated with LLI compensations, Levangie attempted to quantify pelvic asymmetry in a loaded (standing) position without x-ray by using precise location of anatomic landmarks [31]. The objective was to see if pelvic torsion – the most common compensation for LLI – was correlated with back pain. It was not. In another study, a pelvic level – a device with a weighted gravity line superimposed on a scale in one-degree increments clamped in place on the palpated superior aspects of the iliac crests – was used to examine a group of non-clinical subjects [32]. There was no correlation of self-reported back pain, frequency or severity, to pelvic unleveling. However in those subjects with measurable pelvic unleveling (29 of 64 subjects), 61% had a high left iliac crest, which may be evidence of the greater incidence of a longer left leg [32]. A final study, using radiography to determine pelvic obliquity, examined subjects with (n = 93) and without (n = 76) chronic low back pain (defined as low back pain of at least 3 months) [33]. This study found no difference in the pelvic obliquity between subjects with and without chronic back pain, obliquity was prevalent and equally distributed in both groups.

These studies examining pelvic obliquity indicate that this type of postural distortion, be it from LLI or bony asymmetry, is not related to back pain, and does not seem to be clinically significant. The next, more difficult and controversial question is, what is the clinical significance of LLI, and at what magnitude?

### How much anatomic LLI is clinically significant?

Mannello remarked that the clinical significance of LLI was "perhaps...dependent on several factors, including the degree of inequality, the ability of the pelvis and spine to compensate for the inequality and associated conditions or problems" [1]. While this statement is undoubtedly true, this paper will attempt to quantify what ranges of anatomic LLI are clinically significant, that is, being associated with back pain, injury, muscle strength asymmetry or other physiologic changes. Unless noted, all the studies reviewed here have been selected because they used the more accurate radiological methods to determine anatomic LLI.

When one examines references alluding to the clinical significance of anatomic LLI, Friberg's 1983 study [7] is most often cited. Friberg collected data on 1,157 subjects; 798 with chronic LBP and a control group of 359 with no LBP. The data Friberg collected on the prevalence of LLI in a normal population is very similar to that found in the compilation outlined in this paper. The prevalence of LLI 10 mm or greater was 15.6%. This review found the figure to be 14.8%; Friberg showed the incidence of LLI 15 mm or greater at 2.2%, this review 2.6%. Unlike the population compiled in this review however, Friberg's data were obtained from patients at a military hospital and represent a high percentage of subjects exposed to extreme and repetitive loading.

Friberg also reported "LLI was 5 mm or more in 75.4% of the patients with LBP and 43.5% of the controls. The difference is statistically significant (P < 0.001) using a Chi-squared test" [7]. Anatomic LLI greater than 20 mm was previously shown to be the putative limit for spontaneous compensation of the pelvis to postural asymmetry. If these subjects are eliminated from Friberg's data, the association of anatomic LLI with LBP drops somewhat.

**Figure 5:**
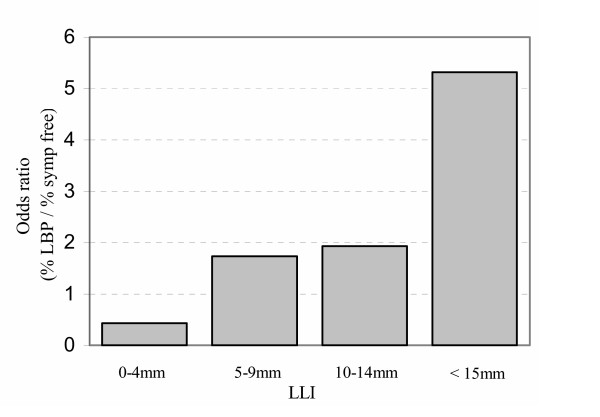
"Incidence" of chronic low back pain with anatomic leg-length inequality (Friberg).

Chronic low back pain (CLBP) affects about 21% of the population [34,35]. One would expect this percentage to be higher if, as Friberg found, that 5 mm of LLI is a causative factor, given that 50% of the population has LLI of 5.2 mm or greater. Figure 5 shows the relative "incidence" of chronic low back pain to LLI using Friberg's data. As can be seen, Friberg's putative correlation really becomes demonstrable when LLI is above 15 mm, at 5.3 times the prevalence of CLBP.

In defending the results and their interpretation in a letter-to-the-editor, Friberg wrote, "... I have always pointed out that LLI of less than 5 mm has no relationship with lumbar scoliosis or back pain. I have also emphasized that even marked LLI *per se *[emphasis in original] neither produces LBP nor contributes to its development if a person is not continually exposed to prolonged standing or gait, e.g., during daily work, military training, and sporting activities" [36]. So, Friberg notes that relatively small amounts of anatomic LLI may only be clinically significant relative to certain conditions such as prolonged and/or repetitive loading – which describes the population in Friberg's study – and not as a generality, as the study is often referenced to support.

Friberg's data represents the low end of anatomic LLI that is hypothesized to be clinically significant. At the high end, in a review of the biomechanical implications of leg-length inequality, others write that LLI less than 30 mm is mild and the clinical significance questionable [25,37]. This large range – from 5 mm to 30 mm – is the likely reason behind the lack of consensus as to the clinical significance of LLI. The answer presumably lies somewhere in between.

Giles and Taylor [30] reported that LLI of 10 mm or larger was found significantly more often in a group with chronic low back pain. No data was given as to the mean LLI or the distribution in the CLBP group, only that the LLI was greater than 9 mm. They found LLI of 10 mm or more in 18% of the CLBP population (n = 1309), and only 8% of the normal population (n = 50). The pooled data (n = 164) of asymptomatic subjects in this review [8,10,12-15] finds 15.5% of this population with LLI of 10 mm or greater. The data compiled from all pooled studies – both symptomatic and symptom free – shows LLI of 10 mm or greater in 15% of the population. These results raise questions about whether the prevalence of LLI found in Giles and Taylor's normal population is representative and whether CLBP is indeed related to LLI in the 10 mm range.

Similarly, Kujala et al found athletes with patellar apicitis (jumpers knee) had a significantly larger LLI (5.8 mm, SD 4.5) than an asymptomatic control group (3.0 mm, SD 2.3) [38]. The mean LLI in the Kujala et al control group (n = 20) is significantly less than the pooled asymptomatic subjects (n = 164) in this review (5.2 mm, SD 4.2) [8,10,12-15] and may be related to the smaller sample size, or the unique group sampled ("healthy" athletes). Regardless, the Kujala et al control group does not represent the asymptomatic general population based on the evidence examined in this review paper. Kujala et al also studied military conscripts (n = 32) who developed knee pain during their initial 8-weeks of training and compared them to a group that did not develop knee pain (n = 28). Those who had knee pain had a significantly larger LLI; 8.0 mm (SD 5.9) versus 4.1 mm (SD 2.9) at p = 0.003 (t-test) [39]. While the magnitude of LLI in the control group in this study is much closer to the normal demonstrated in this review paper (5.2 mm versus 4.1 mm), the magnitude of the control group LLI is also very close to that of the patellar apicitis group in the athlete study. One might question why athletes are more likely to develop knee pain with an average LLI of 5.8 mm, but training soldiers with an average LLI of 4.1 mm are not? Further, there was no correlation between the injured knee and the side of the short leg, which would be expected if the short leg were the predisposing factor.

In a survey of 247 working age men and women looking for the presence of LLI, Soukka et al [13] examined and compared statistically matched groups with and without LBP. Their results showed no increased risk of back pain with a LLI of 10–20 mm, and the relationship between LLI of more than 20 mm and back pain were not conclusive. These results differ markedly from that of Friberg, prompting the letter-to-the-editor noted above [36]. In the exchange between Friberg and Soukka et al, both agree that the significance of LLI may depend more on prolonged and repetitive loading, a common sense idea previously expressed by Subotnick [40].

One of the areas of research into the clinical significance of LLI has been in relation to femoral fracture and total hip replacement surgery. Gibson et al found that in 15 patients, at least 10 years after shortening due to femoral fracture (average 3 cm, range 1.5 – 5.5 cm), there was no significant discomfort, structural abnormalities or degenerative changes as a result of the leg length discrepancy [41]. Edeen et al followed 68 patients with a mean LLI of 9.7 mm for an average of 6.6 years after hip replacement surgery [42]. They were not able to demonstrate a relationship between LLI and low back pain. Another study of 200 post total hip replacement surgeries used validated functional outcome scores (Harris hip score and the SF-36 Health Survey) to examine the relationship of imposed LLI to functional outcome [43]. This study found that leg lengthening (up to 35 mm) or shortening (up to 21 mm) did not correlate with decreased function, comfort or satisfaction six months after the operation. A retrospective study of 6,954 total hip arthroplasty patients over a 7 year period found only 21 (0.3%) had symptoms related to post-surgery leg length inequality symptomatically severe enough (primarily back and hip pain) to require a second surgery to equalize leg length [44]. The mean LLI of the patient's who received revision arthroplasty was 3.6 cm (± 1.2 cm, range 2.0 cm to 7.0 cm). The results of these studies of hip replacement are somewhat surprising given that the LLI was induced at an older age when the ability of the pelvis, SI joints and soft tissues to compensate for this asymmetry would likely be reduced.

In examining the effects of LLI from childhood, Yrjönen et al [45] did a follow-up study of 81 patients with Perthes' disease and a mean LLI of 12 mm. The follow-up time was an average of 35 years (range 28–47). They found that most of the patients had no back pain, and concluded that back pain was not a significant problem after Perthes' in spite of frequent LLI. Another study of adults (mean age 28) with large LLI since childhood – mean 29.1 mm – found no complaints of back pain or degenerative changes. Lumbar scoliosis was minor in those with LLI of less than 22 mm [46].

In most of these studies, follow-up was years to decades, and LLI means from ~ 10 mm to 30 mm, yet none could demonstrate a significant correlation to back pain. Given these findings, the average 5 mm anatomic leg-length differential does not appear to be significant, even with prolonged and repetitive loading. Based on these studies, childhood-onset LLI up to at least 20 mm (~ 3/4") does not seem to be clinically significant.

Another category where LLI can cause sudden, abnormal loading of the lumbar-pelvic structure is in athletic and military training. Gross [10] examined LLI in a group of marathon runners. He found that leg length discrepancy less than 25 mm did not appear to have a deleterious effect. In a study of stress fracture and LLI in Finnish army conscripts, Friberg [47] found those with LLI ≥ 10 mm had stress fractures 10% more frequently than healthy (no known stress fracture) controls. No statistical analysis was described, so it is not known whether the increase in stress fracture incidence from 20.1% (controls) to 30% (patients) is significant. Friberg did find that in parachutists, those with LLI 10 mm or more (15.7% of 102, n = 16), 50% had stress fractures. This does point to an association between LLI of over 10 mm, extreme loading and stress fracture, however, the small "n" of 16 did not allow for a statistical analysis. In a study of athletes (n = 46) for anatomic LLI as a risk factor in stress fractures, Korpelainen et al [48] found the mean LLI of the patient group to be 4.9 mm. While sympathetic to the possibility of the association between LLI and stress fracture, they found no relationship.

Again, the average amount of LLI (5 mm) does not appear to be clinically significant with substantially increased and repetitive loading. Only when the increased loading is abrupt and severe (Friberg's parachutists) is a strong correlation established between LLI of 10 mm and a pathologic condition (stress fracture). Given the findings in these studies, LLI below 10 mm, even with heavier repetitive loading, does not appear to be clinically significant. LLI between 10 – 20 mm increases the chances of clinical significance, but outside of severe, abrupt loading, the evidence is lacking. Based on these studies, it would appear that childhood-onset LLI of up to 15 – 20 mm does not seem to be clinically significant.

The effect of LLI on physiological function has also been explored, and can shed some light on a possible range of clinical significance. It has been presumed that anatomic LLI, because of its effects on structure, causes muscular hypertonicity and changes in strength and/or coordination [28]. Mincer et al [15] expected LLI, (because of presumed stressful mechanical effects on the lumbar spine by virtue of the asymmetrical loading) would cause earlier and greater fatigue of trunk muscles, and tested that hypothesis. The average inequality in the LLI group (n = 18) was 10 mm. They found no difference between the LLI and no LLI groups relative to muscle fatigue or neuromuscular control. Yen et al [49] examined muscular performance on trunk extension in a group of young men with estimated LLI of 10 – 15 mm, both with and without a lift used to equalize LLI. There was no statistically significant effect of the lift and equalization of LLI on any of the variables tested. Murrell et al [50] examined standing balance in subjects with LLI of at least 9.5 mm versus those with no LLI and found no difference. They concluded that individuals with anatomic LLI are not less stable than those without during quiet stance, and that the probable reason for this finding is long-term adaptation by the neuromuscular system to the LLI.

The last two studies [49,50] relied on more inaccurate measures to determine LLI, so the results are suspect. However, in these studies, care was taken to classify and examine only those with far end-range amounts of asymmetry as having LLI; in all studies that amount was over 10 mm.

In a study of LLI and analysis of gait, Goel et al found no significant differences in joint movement with the imposition of a 1.25 cm leg length differential via a shoe (not just heel) lift [51]. Based on their findings, they suggest that, "...the body is well able to compensate for minor LLD [leg length differential] of up to 2 cm. Correction of an LLD of this magnitude for biomechanical reasons alone does not appear indicated". Another study of gait with LLI imposed via foot lifts found that a 2.3 cm lift produced no changes in gait or hip forces and moments [52]. A study of subjects with pre-existing LLI found that a mean LLI of 2.5 cm was necessary to produce an asymmetrical gait [53]. A study of the effect of LLD in children (n = 20, 9.0 ± 3.9 years) on found gait asymmetries only with LLD >2.0 cm [54]. White et al found LLD between 1 – 3 cm, whether simulated or real, resulted in unequal loading of limbs when walking, and recommended considering shoe lifts to equalize leg lengths [55]. Finally, in examining the effects of imposed foot lifts on oxygen requirements, one study found no statistical difference even with a lift of 3 cm during running [37]. Another found it was necessary to impose a LLI of between 2 – 3 cm in older adults in order to cause increased oxygen consumption and perceived exertion [56].

As in the previous groups (general working population, long-term loading, and heavy loading) the effects of LLI in the order of 10 mm relative to muscle strength, coordination and gait and oxygen consumption do not appear clinically significant. The evidence in these studies is less compelling because of the measurement methods, the concentration of testing around the 10 mm mark and imposition of LLI, which does not give the body time to compensate. However, there is no reason to believe that those physiological measures are any more sensitive to LLI than the other measures noted previously.

These findings – that LLI in the range of 20 mm (~ 3/4"), regardless of prolonged or repetitive loading, does not result in back pain or other clinically significant symptom, seems to preclude the need for heel lifts in most cases. However, there will always be individual exceptions, and there may be some general exceptions.

Gofton and Trueman found a strong association between leg length and unilateral osteoarthritis (OA) in the supero-lateral region of the hip on the side of the anatomically long leg [4]. In their study, all subjects with this type of OA "had led healthy active lives prior to the onset of hip pain", and few subjects were aware of any difference in leg length. The authors point out that this form of OA has its onset around the age of 53. While concluding that anatomic LLI in the order of 1/2" to 1" (13 – 25 mm) is associated with the development of a unilateral OA hip, Gofton and Trueman acknowledge that many with this anatomic asymmetry fail to develop this condition, suggesting that factors other than the disparity are also important. An important area of investigation would be to determine these other factors to provide a clearer picture of who may be at risk.

Further data suggesting exceptions to the conclusions drawn above regarding the effects of mild anatomic LLI come from Triano [57]. He demonstrated balancing of asymmetric electromyographic paraspinal muscle activity in 51% of subjects with low back pain by using an average heel lift of 22 mm. These results indicate that changes in leg length of ~3/4" or greater results in active – muscular – compensation which, if prolonged, may become painful. Bringing the pelvis back towards a neutral orientation and decreasing active muscular compensation may explain why the use of heel lifts under the short leg appears to be an effective treatment in some complaints of back pain [7,16,58]. To explain these results, the functional "short leg" will be examined in Part II.

In summary, childhood-onset anatomic leg-length inequality appears to have little clinical significance up to 20 mm. Several authors agree [2,25,59], most recently with Kakushima et al who stated: "Therefore, although conflicts in the literature exist, 3 cm of LLD [leg length discrepancy] can be characterized as a minimum LLD, which should be treated in the clinical practice" [60]. This estimation of clinical significance dovetails nicely with the findings on the effects of LLI, particularly pelvic torsion [23]. Passive structural changes – pelvic torsion, mild lumbar scoliosis, facet angulation, changes in muscle length – seem capable of compensating for anatomic LLI of up to 20 mm. Past the ~ 20 mm point, passive structural changes give way to active muscular compensatory measures.

## Conclusion

The purpose of this paper was to review radiographic research regarding anatomic leg-length inequality; prevalence, mean magnitude, effects, clinical significance and relationship to unloaded leg-length alignment asymmetry. Ninety per cent of the population has some anatomic leg-length inequality; the average was found to be 5.2 mm. Based on the research reviewed, childhood anatomic LLI of less than 15 mm in situations of repeated and/or heavy loading, or less than 20 mm (~ 3/4") under normal conditions, is not likely to cause symptoms requiring treatment.

## Competing interests

The author(s) declare that they have no competing interests.
